# Uncertainty in the timing of diversification of flowering plants rests with equivocal interpretation of their fossil record

**DOI:** 10.1098/rsos.242158

**Published:** 2025-05-28

**Authors:** James W. Clark, Philip C. J. Donoghue

**Affiliations:** ^1^ Milner Centre for Evolution, Department of Life Sciences, University of Bath, Bath BA2 7AY, UK; ^2^ Bristol Palaeobiology Group, School of Earth Sciences, University of Bristol, Bristol BS8 1TQ, UK

**Keywords:** angiosperm, timescale, fossil record, molecular clock, macroevolution, flower

## Abstract

The timing of the origin of crown-angiosperms exemplifies the impact of competing approaches to establishing evolutionary timescales. Fossils of unequivocal crown-angiosperms are not known from before the Cretaceous, and yet molecular estimates range from the Late Jurassic to the Permian. We show that the disagreement between molecular and palaeobotanical estimates is an artefact of interpretations of the fossil record. We employ relaxed molecular clock methods that reflect competing interpretations of the fossil record to show that such methods are entirely capable of recovering an explosive diversification of angiosperms if the fossil record can be interpreted confidently to support this. We argue that older putative angiosperm records have insufficient claim on crown-angiosperm affinity to justify their use in divergence time estimation and, in their absence, estimate crown-angiosperms to have diverged in a Late Jurassic–Early Cretaceous interval. This diminishes the Jurassic gap between molecular clock estimates and literal interpretations of the fossil record but expands the Jurassic gap in the fossil record of stem-angiosperms that is not readily rationalized. Attention should be refocused on the history of stem-angiosperms in which the body plan of this most successful lineage of land plants was assembled.

## 1. Introduction

**Figure 1.**
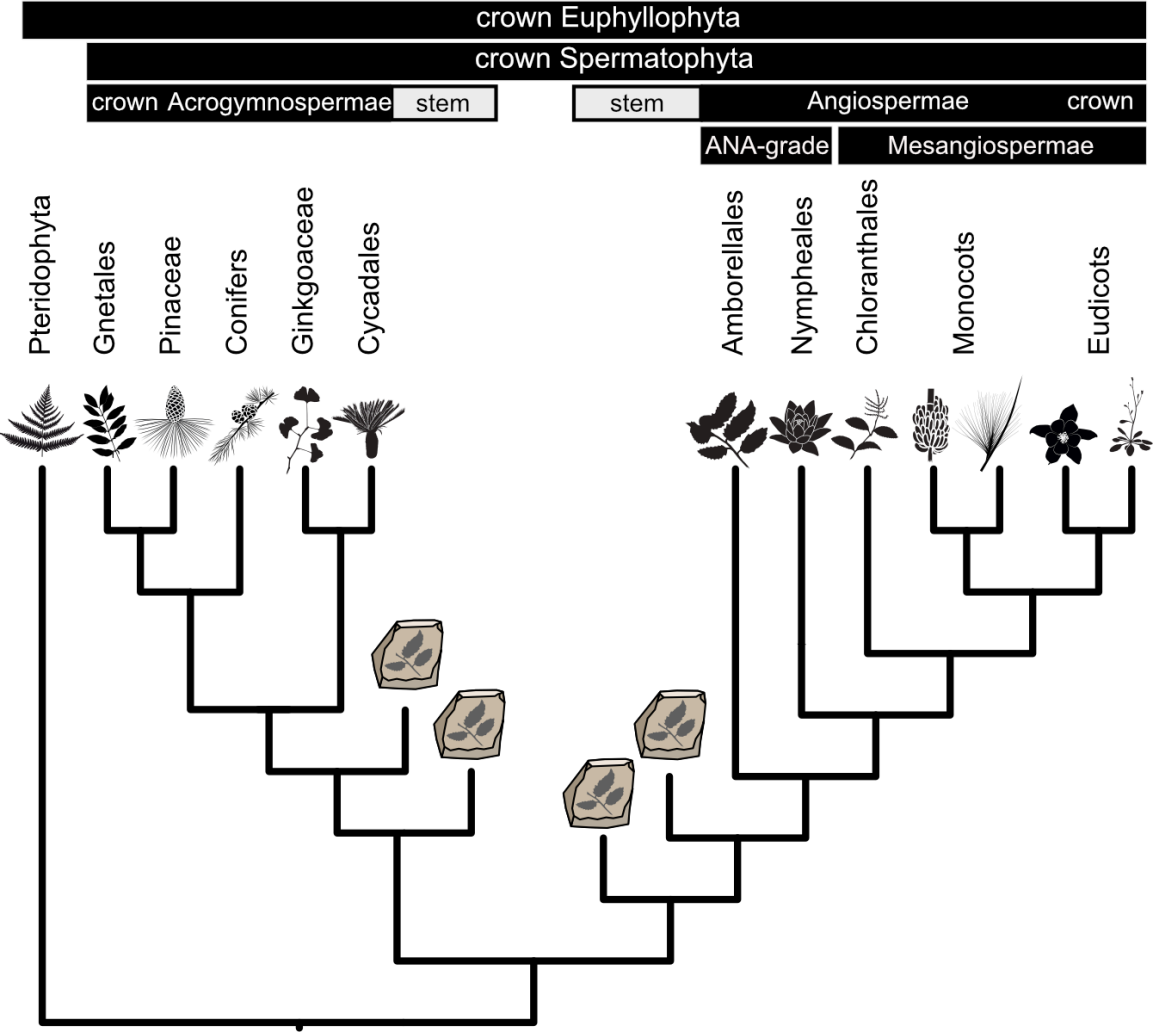
Clade terminology referred to in this study.

Angiosperms (flowering plants; see figure 1 for clade terminology) are the most diverse of all land plant lineages, the explosive radiation of which appears to have precipitated the diversification of many other lineages of plants, animals and fungi [1]. Genome sequencing and advances in the modelling of phylogenetic data have yielded greater insights into the underlying conflicts and processes in angiosperm phylogeny [2–4], but, despite robust debate, their evolutionary timescale remains enigmatic [5–8]. Varying timelines of angiosperms are frequent both between and within analyses, and this is no more apparent than in Zuntini *et al*. [4], who presented one of the most densely sampled phylogenomic studies of angiosperms to date, yet were still required to present two competing and wholly incompatible evolutionary timescales because of uncertainty associated with molecular clock calibration. Elsewhere, accommodating multiple calibration schemes results in estimates that span differing interpretations, resulting in inclusive yet imprecise estimates [9,10].

Reports of pre-Cretaceous angiosperm macroremains have largely been rejected as gymnosperms, though some records are excluded because they do not preserve unequivocal crown-angiosperm characters [5,6,8,11]. Nevertheless, it is clear that crown-angiosperms have a deeper evolutionary history than their macrofossil record suggests. This is evidenced by the pollen record that exhibits a coherent relationship between the stratigraphic order of appearance of evolutionary grades and the phylogenetic branching order of clades [5], beginning with the appearance of monosulcate pollen of the early diverging ANA-grade plants (*Amborella*, Nympheales and Austrobaileyales), magnoliids, Chloranthales and monocots, followed by the tricolpate pollen of early branching eudicots and then the tricolporate pollen grains of more derived eudicots [5]. This record begins in the Valanginian (Early Cretaceous, specifically approximately 136 Ma) and, given the almost refractory nature of their sporopollenin composition, the pollen record has reasonably been interpreted to evidence a Cretaceous origin of crown-angiosperms.

The angiosperm crown-ancestor must be older than the oldest unequivocal fossil record of a clade, if only because lineage divergence is a phenomenon of the genome that can only be diagnosed in the fossil record when derivative lineages evolve fossilizable apomorphies [12]. Hence, Coiro *et al*. [5] reason that the interpretation of the pollen fossil record probably requires the latest Jurassic origin. Molecular clock methodology has been applied to constrain the extent of this mismatch between fossil minima and clade age (reviewed in [7]). Some molecular estimates are more than 100 Myr older than the earliest unequivocal angiosperms [13], but others suggest that the mismatch could be as little as 23 Myr [9,10,14]. It has been argued that this temporal disparity is a consequence of systematic biases in molecular clock methods, despite their diversity, particularly in terms of the underlying rate and birth–death models, as well as the specification of the effective prior [15–18]. However, the most significant variable in molecular clock analyses is their calibrations, which have been developed in recent years to eschew uncertain fossil evidence in establishing minima but establishing maxima to encompass older but less certain fossil records of clades (e.g. [19–22]). As such, while calibrations have been developed to encompass the diversity of interpretations of the fossil record, they have become less informative, using clock methods to arbitrate among the diversity of opinions over how the fossil record should be interpreted.

We explore the impact of more decisive interpretations of the fossil evidence, specifically in establishing less permissive maximum bounds on fossil calibrations in molecular clock analyses. The key variables here are equivocal records of crown-angiosperms that have been called into question because they are temporally isolated from the rest of the crown-angiosperm fossil record and do not preserve or did not possess unequivocal crown-group synapomorphies [5,6]. These are *Phyllites* sp.*,* known from poorly preserved leaves from the Middle Jurassic of Oxfordshire that exhibit a venation pattern characteristic of crown-angiosperms [23,24] and records of crown-angiosperm-like pollen from the Anisian (Middle Triassic [25,26]) of Switzerland, Norway and Italy. Otherwise, the maximum bound on the angiosperm crown is based on the oldest records of their sister lineage, the acrogymnosperms, which date back to the Late Carboniferous [22,27]. We show that alternative interpretations of the fossil record and, in turn, calibration strategies have an overwhelming impact on estimates of the age of angiosperm crown. Importantly, this demonstrates that there is no methodological bias against recovering divergence time estimates that are in close agreement with different interpretations of the fossil record. Relaxed molecular clock methods are quite capable of accommodating the dramatic shifts in the rate of molecular evolution required by an explosive Cretaceous origin of crown-angiosperms. However, analyses have not recovered such timescales because the fossil record, as currently interpreted, has not generally been deemed sufficiently informative to justify the calibrations required.

## 2. Material and methods

### 2.1. Assembly of a phylogenomic dataset

We sought to broadly sample angiosperm lineages while also maximizing the number of reliable fossil calibrations. For tractability, we subsampled 93 species from a recent phylogenomic dataset [10] that initially comprised 644 species, preserving the diversity of extant angiosperms and retaining 10 non-angiosperm outgroup species (electronic supplementary material, figure S1, table S1). The reduction in species sampling was designed such that each of the fossil calibrations employed in the original study was preserved at the same node in the revised dataset, totalling 52 calibrations (electronic supplementary material, figure S1, table S2). The dataset includes markers from the nuclear (5081 nucleotides), mitochondrial (5690 nucleotides) and plastid (41 021 nucleotides) genomes and is partitioned by subgenome. Further, the third codon position in protein-coding genes was removed to reduce the impact of sequence saturation. Barba-Montoya *et al*. [10] compared parameter specifications across multiple analyses but ultimately concluded that a relaxed clock (independent lognormal; IL) model fits the data best, where rates for each branch are independently drawn from a lognormal distribution. This corresponds to the ‘SA-IR-3P’ dataset of [10] in which the independent rates model was applied to the data across three partitions. The full dataset is available in the electronic supplementary material.

### 2.2. Molecular clock analyses

All molecular clock analyses were run using codeml and mcmctree in PAML (version 4.9.j) [28] on a fixed topology as recovered by [10]. We used the normal approximation method, where the branch lengths and Hessian matrix are first estimated, in this case for each partition under the HKY+G4 model, and these branch lengths are used to inform the molecular clock. Calibrations were specified either as a uniform distribution between a minimum age and a maximum age constraint (reflecting ambivalence concerning the true clade age, per unit time, between these bounds), plus a 2.5% soft tail distribution added to the maximum age constraint. Calibrations, where a maximum age is not specified, were modelled as a truncated Cauchy distribution [29], with parameters *p* = 0.1, *c* = 0.1 and pL = 0.01 representing the offset (distance of the mode from the minimum), scale (rate of decay) and probability of minimum age being exceeded. These values, following [10], represent a scenario where the mode of the probability occurs close to the minimum and rapidly decays. The parameters of the clock model followed the original analyses. The rate for each branch is drawn from a lognormal distribution with mean *μ* and variance 𝜎^2^. The values for *μ* and 𝜎^2^ were modelled as a gamma prior with shape and scale parameters of 2 and 50 for *μ* and 2 and 5 for 𝜎^2^. The birth and death parameters of the tree model were each set to 1, specifying a uniform kernel. Input files for each analysis are available in the electronic supplementary material. All analyses were run with four independent chains for 10 million generations, with the first 25% of each run discarded as burn-in.

## 3. Results

### 3.1. Molecular clock calibrations

We established fossil calibrations following best practice principles [30]. We used the same suite of calibrations implemented in [10], with the exception of the calibration on the crown-angiosperm node. For this, we kept the minimum age constraint constant and explored the impact of competing maximum constraints based on alternative interpretations of the fossil record. In all instances, the minimum constraint on the age of the crown-ancestor of angiosperms was defined as:

**Fossil taxon and specimen**. Tricolpate pollen grain [palynological sample BRN 126] from the middle Atherfield Wealden Bed 35 of the Cowleaze Chine Member, Vectis Formation, Barremian (Early Cretaceous), of the Isle of Wight [31].

**Phylogenetic justification**. Following [22], our minimum age constraint is based on the earliest occurrences of Fischer’s rule of tricolpate pollen and knowledge of the distribution of tricolpate pollen across the phylogeny of angiosperms [32].

**Minimum age**. 125.9 Ma.

**Minimum age justification**. Following [22], the soft maximum age constraint is based on the maximum age of the oldest potential age of tricolpate pollen from the middle Atherfield Wealden Bed 35 of the Cowleaze Chine Member, Vectis Formation.

**Figure 2.**
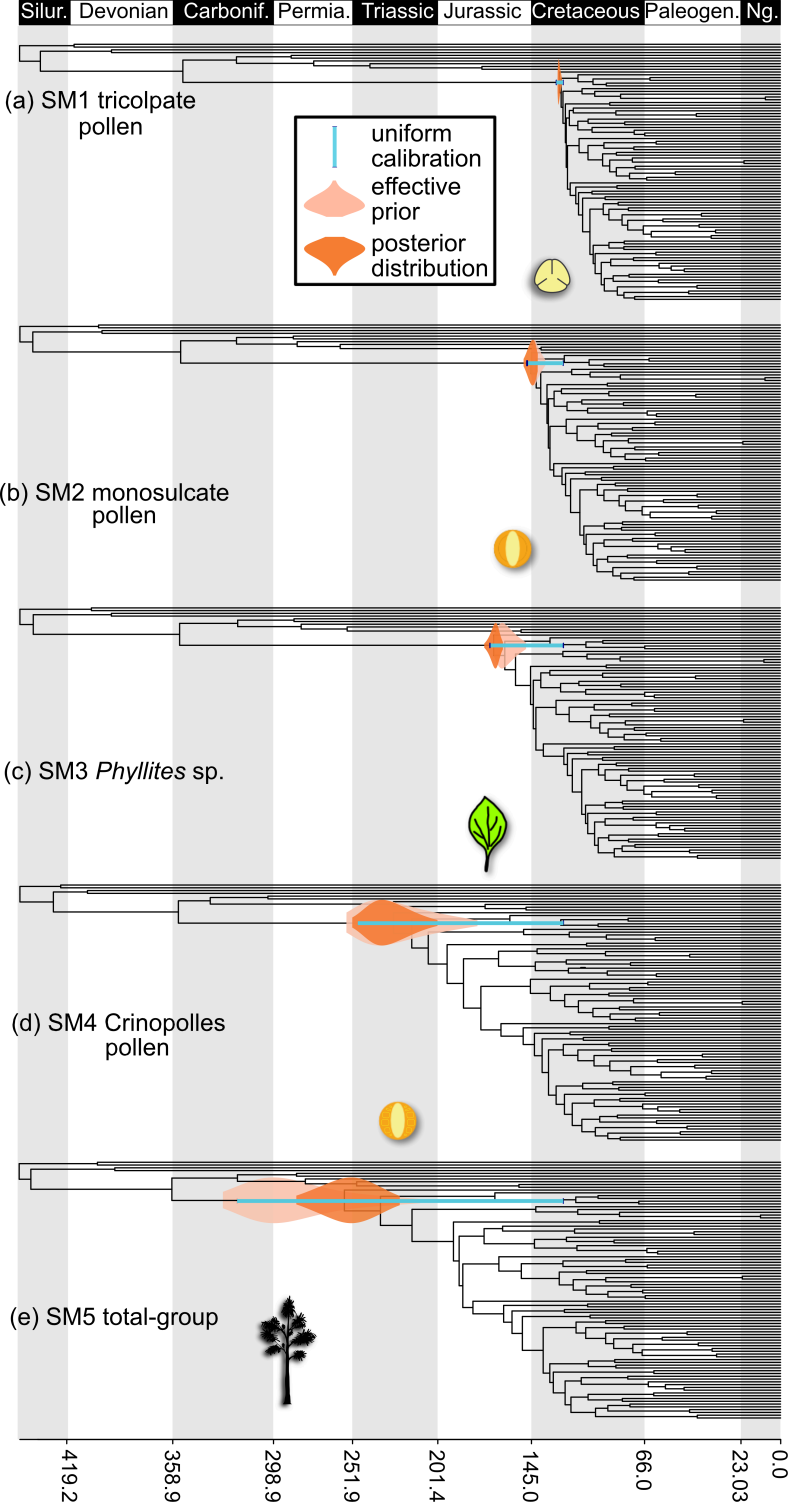
The inferred age of crown-group angiosperms under different maximum age calibrations. For each calibration, the effective prior and posterior age distribution is plotted as a violin, with the specified uniform calibration as a bar.

**Specified, effective priors and posteriors**. The constraint on the maximum age of crown-angiosperms was varied according to the five following possible soft maxima, each reflecting a different interpretation of the oldest plausible evidence of the crown-angiosperm lineage in the fossil record. In each instance, these maxima were augmented with a 2.5% probability tail to allow for the fact that the oldest phenotypic evidence of divergence must post-date genomic divergence (figure 2).

### 3.2. Soft maximum 1: Barremian tricolpate pollen

The maximum age is based on the same specimen used to establish the minimum age constraint, given that this represents the oldest unequivocal record of the angiosperm crown group.

**Fossil taxon and specimen**. Tricolpate pollen grain [palynological sample BRN 126], from the middle Atherfield Wealden Bed 35 of the Cowleaze Chine Member, Vectis Formation, Barremian (Early Cretaceous), of the Isle of Wight [31].

**Soft maximum age**. 128.63 Ma.

**Soft maximum** **age justifi****cation**. Following [22], the Cowleaze Chine Member of the Vectis Formation of the Isle of Wight [31] occurs within the M1n polarity chron in the Late Barremian. The base of the M1n chron is approximately correlated with the base of the *Gerhardtia sartousi* biozone, dated at 128.63 Ma.

**Discussion**. This calibration effectively assumes that the earliest unequivocal record of crown-angiosperms equates to the time of origin of the clade. It differs in age from the minimum constraint only in terms of the different procedures for establishing maximum versus minimum constraints, which is to take the oldest rather than the youngest age interpretation of the fossil record. Thus, the span of the minimum–maximum constraints is 2.73 Myr, which makes it very informative but potentially inaccurate since it excludes older, Valanginian, records of monosulcate pollen that are generally accepted to be derived from ANA-grade non-mesangiosperm angiosperms [5].

**Specified and effective priors**. Under this calibration, the effective (joint-time) prior predicts an origin for crown-angiosperms within the Early Cretaceous, marginally exceeding the specified calibration (130–126.3 Ma; Hauterivian–Barremian) (figure 2).

**Posterior clade age estimate**. Combined with sequence data, the posterior estimate is very precise, inferring an origin for crown-angiosperms during the Early Cretaceous (129.4–130.5 Ma, Hauterivian) that exceeds the specified prior. This scenario provides the narrowest window for the diversification of the mesangiosperm lineages, with the magnoliids, monocots and eudicots all diverging within 8–6 Myr following the origin of crown-angiosperms (figure 2).

### 3.3. Soft maximum 2: Valanginian monosulcate pollen

**Fossil taxon and specimen**. Monosulcate pollen grain, Helez Formation, lower sand member, Kokhav 2 well, core 5, Late Valanginian/Early Hauterivian [33].

**Phylogenetic justification**. The maximum age is derived from the earliest monosulcate pollen grains that are unequivocally angiospermous [33]. Monosulcate pollen is the ancestral state in angiosperms [34], but the affinity of these pollen specimens as either crown or stem angiosperms is not resolved.

**Soft maximum age**. 142.8 Ma

**Soft maximum age justification**. The soft maximum age constraint is based on angiospermous pollen found in the lower sand member of the L.CrIIa unit of the Helez Formation, Israel [33]. The lower sand unit is determined as Late Valanginian–Early Hauterivian, based on the presence of Early Hauterivian ammonites in the upper part of the L.CrIIa above the lower sand member and Valanginian ostracods and foraminifera below it [33]. The calibration is thus derived from the earliest sediments containing angiosperm-like pollen, excluding reports from the Middle Triassic, in the Valanginian, dated to 139.8 ± 3 Ma, thus 142.8 Ma [35].

**Discussion**. Although these monosulcate pollen records are widely accepted to be of angiosperm grade, it is not known whether this grade of pollen is limited to the crown-group or whether it is a characteristic of the angiosperm stem-lineage as well. As such, it is quite possible that calibrations based on these earliest records of monosulcate pollen reflect the age of a more inclusive clade than crown-angiosperms. However, the span of the minimum–maximum constraints is still only 16.9 Myr, and so if it does encompass some older stem-angiosperm records, the calibration remains very informative. Perhaps the greatest concern is that it excludes the older record of *Phyllites* sp. from the Middle Jurassic Stonesfield Slate [23], a fossil leaf that has an angiosperm-grade venation pattern.

**Specified and effective priors**. Under this scenario, the effective prior again marginally exceeds the specified maximum age and proposes an origin of crown-angiosperms within either the Early Cretaceous or Late Jurassic (148.7–133.9 Ma; Tithonian–Valanginian) (figure 2).

**Posterior clade age estimate**. The posterior proposes a more ancient origin again, from the Late Jurassic to the Jurassic–Cretaceous boundary between 155.3 and 145.0 Ma (Tithonian–Kimmeridgian), with no overlap between the posterior and the specified prior (figure 2).

### 3.4. Soft maximum 3: *Phyllites* sp.

**Fossil taxon and specimen***. Phyllites sp.* [NHM 41385 and V.85, Natural History Museum, London, UK] from the Middle Bathonian, Stonesfield, Oxfordshire, UK [23].

**Phylogenetic justification**. The angiospermous nature of the leaf is suggested based on the ovate shape and reticulate and actinodromous or acrodromous (palmate) venation [5,23,24,36]. This pattern of venation indicates a position within crown-angiosperms, and character reconstruction has suggested that palmate venation is a derived character present in some Nympheales, Piperales, Laurales or eudicots [37].

**Soft maximum age**. 167.3 Ma.

**Soft m****aximum age ju****stification***. Phyllites* sp*.* was collected from the Stonesfield Slate, Oxfordshire, UK, a lithofacies of the Taynton Limestone Formation [38]. Following the ammonite fauna, the Taynton Limestone Formation is referred to as the *Procerites progracilis* biozone, which corresponds to the lower part of the Middle Bathonian [38,39]. The base of the *Procerites progracilis* biozone is dated 167.37 Ma [40].

**Discussion**. This is a credible candidate for a pre-Cretaceous angiosperm macrofossil. It is not well preserved, but it exhibits an acrodromous venation; preservation is too poor to discriminate the venation architecture further [37], but what is preserved is compatible with crown angiosperm affinity [37] since palmate venation patterns are considered a derived (and convergent) character of Nymphaeales, Piperales and eudicots [41]. Indeed, Seward [23] wrote, ‘Had the specimen been found in rocks known to contain the remains of Angiosperms, there would be no hesitation in identifying it as a leaf of a Dicotyledon’. Cleal and Rees [24] have confirmed that the sediment in which *Phyllites* sp. is preserved is petrologically indistinguishable from the rest of the flora and fauna of the Stonesfield Slate. However, *Phyllites* sp. suffers from its poor preservation and what might be characterized as ‘the early angiosperm uncertainty principle’ in that it is divorced temporally from other macrofossil records of angiosperms, which has been sufficient for many to doubt its crown-angiosperm affinity. It has also been observed that broad leaves and angiosperm-like venation patterns may arise convergently in other seed and non-seed plants [42], including Peltaspermales [43], and so convergent evolution of leaf form and venation cannot be excluded. *Phyllites* sp. expands the span of the minimum–maximum constraints by 41.4 Myr, making for a more uninformative crown-angiosperm calibration, and it is questionable whether the quality and quantity of evidence can bear the burden of a 24.5 Myr extension on this uncertainty relative to soft maximum 2.

**Specified and effective priors**. While the specified prior is 167.3–125.9 Ma (plus a 2.5% soft tail distribution on the maximum), the effective prior is 168.6–149.5 Ma (Middle to Late Jurassic) (figure 2).

**Posterior clade age estimate**. The posterior estimate is 174.7–163.5 Ma (Middle Jurassic) (figure 2).

### 3.5. Soft maximum 4: Triassic (Anisian) monosulcate pollen

**Fossil taxon and specimen***. Retisulcites* from the Steinkobbe Formation, Anisian (Middle Triassic) Norwegian Arctic [44].

**Phylogenetic justification**. Pollen types C–F of Hochuli and Feist-Burkhardt [25] from the Snadd Formation, Norwegian Arctic, and type C ‘*Retisulcites*’ from the older Steinkobbe Formation [44] represent the oldest pollen which possess synapomorphy combinations most similar to Cretaceous grains.

**Soft maximum age**. 247.3 Ma.

**Soft maximum age justification.** The soft maximum age constraint is based on sediments devoid of angiosperm-like pollen below their first report in the Middle Triassic. Type C ‘Retisulcites’ of Hochuli and Feist-Burkhardt were recovered from the Steinkobbe Formation, Norwegian Arctic. The Steinkobbe Formation is considered to be Middle Anisian in age [45]; thus, the maximum age of these pollen grains is at the base of the Anisian, dated at 247.1 ± 0.2 Ma.

**Discussion**. Anisian records of angiosperm-like pollen pre-date the Late Triassic *Crinopolles* group monosulcate pollen records of Cornet [46], who considered them reminiscent of Gnetales, supporting his view of a close relationship between Gnetales and angiosperms. Friis *et al*. [47] and Herendeen *et al*. [6] draw a comparison between the range of apertural types exhibited by *Crinopolles* group pollen and that of *Eucommidites,* which, after a history of being compared to crown-angiosperm pollen, have been attributed to the extinct group Erdtmanithecales, which is considered more closely related to acrogymnosperm clades such as Gnetales [48]. The outer exine of the pollen grains consists of an angiosperm-like reticulate tectum supported by columellae; the endexine is thick and unlaminated, indicative of acrogymnosperm affinity. Doyle and Hotton [49] argued that the absence of endexine lamination could be preservational; laminated endexine is absent from the pollen of Bennettitales, which in some analyses have been resolved as stem-angiosperms [50]. Furthermore, the uniform thickness of the endexine is more compatible with gymnosperm pollen since the endexine is generally thin or absent, except at the aperture in angiosperm pollen. Doyle and Hotton [49] and Doyle [37] preferred a stem-angiosperm affinity for *Crinopolles* group pollen, which is the most logical interpretation of the combined gymnosperm (likely seed plant symplesiomorphy) and angiosperm characteristics. The Anisian pollen grains reported by Hochuli *et al*. [25,44] have not yet been subjected to ultrastructural analysis, and so it is unclear whether their characteristics conform to the expectations of *Crinopolles* group grains. All these interpretations of affinity are subject to testing upon discovery of associated macroremains; however, in the interim, they can be interpreted as stem-angiosperms. Magallón *et al*. [14] argued, based on the morphological uncertainty and the 100 million-year gap between these and other angiosperm pollen, for excluding these from calibrations. The inclusion of these Triassic records expands the span of the minimum–maximum constraints to 121.4 Myr. The probability that the Anisian angiosperm-like pollen grains represent crown-angiosperms is non-zero, and so there may be a case for establishing a maximum constraint on the age of crown-angiosperms on the oldest record of their nearest extent sister lineage, the gymnosperms.

**Specified and effective priors**. Under this calibration scheme, the specified prior is 247.3–125.9 Ma (plus a 2.5% soft tail distribution on the maximum) and the effective prior is 251.2–175.8 Ma (Early Triassic–Early Jurassic) (figure 2).

**Posterior clade age estimate**. The posterior estimate is 250.6–201.9 Ma (Early–Late Triassic) (figure 2).

### 3.6. Soft maximum 5: earliest crown-spermatophyte

**Fossil taxon and specimen***. Cordaixylon iowensis* [UIC 12,233, OUPH 9616–9742: Ohio University Paleobotanical Herbarium, Department of Botany, Ohio University, Athens, Ohio] from coal balls from the Laddsdale Coal, Lower Cherokee Group, Desmoinesian (upper Moscovian–Kasimovian), Middle–Upper Pennsylvanian (Carboniferous), near What Cheer, Iowa [51].

**Phylogenetic justification**. Clarke *et al*. [22] identify cordaitelean coniferophytes as the oldest records of the crown group of the Acrogymnospermae clade. The oldest whole plant reconstruction is *Cordaixylon iowensis* from the Laddsdale Coals, Cherokee Group, near What Cheer, Iowa [51]. Acrogymnospermae are the sister lineage to angiosperms, and so their presence confirms the earliest possible age of angiosperms.

**Soft maximum age**. 312.38 Ma

**Soft maximum age justification**. Janousek and Pope [52] argue that the Laddsdale Coal is equivalent to the Bluejacket Coal of Oklahoma, which occurs as part of the Bluejacket Sandstone Member, underlying the Inola Limestone, part of the Inola Cyclothem of the Krebs subgroup of the Cherokee Group, characterized by the occurrence of the conodonts *Idiognathodus amplificus, Idiognathodus podolskensis* and *Neognathodus asymmetricus* [53]. The Inola cyclothem falls fully within the *Idiognathodus amplificus/Idiognathodus obliquus* biozone [54]. This is indicative of the *Neognathodus medexultimus—Streptognathodus concinnus* (Pc10) biozone, certainly older than the *Neognathodus roundyi—Streptognathodus cancellosus* (Pc11) biozone [54]. The base of Pc10 is bracketed by an older age constraint of 312.01 ± 0.37 Ma, yielding a maximum constraint of 312.38 Ma.

**Specified and effective priors**. Under this calibration scheme, the specified prior is 312.38–125.9 Ma (plus a 2.5% soft tail distribution on the maximum) and the effective prior is 323.4–202.1 Ma (figure 2).

**Posterior clade age estimate**. The posterior estimate is 288.4–226.9 Ma (Permian–Late Triassic) (figure 2).

### 3.7. Rates

**Figure 3.**
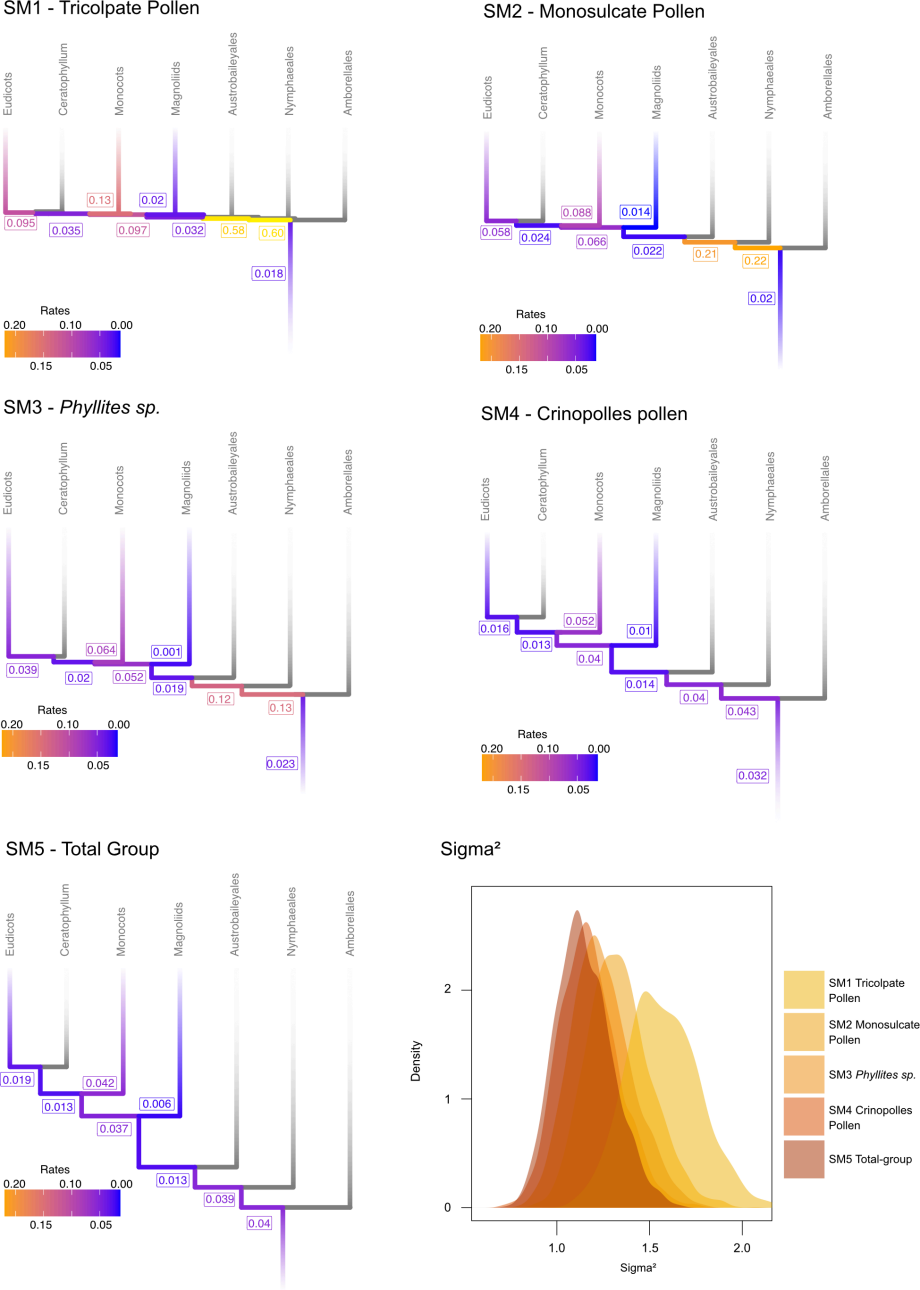
The inferred rate of molecular evolution (substitutions per site per 100 Myr) for the total alignment along key branches under successive calibration schemes. A colour scale indicates the mean rate of molecular evolution along each branch. Variation in the rate of change across branches (bottom right) is modelled by the parameter 𝜎^2^, indicative of how much the clock is violated.

The inferred rate of molecular evolution along each branch and for each molecular partition was estimated under each calibration scheme. The overall clock rate (*μ*) for all three partitions was highest under SM1 and lowest under SM5 across all partitions, with SM1 roughly twice as fast as SM5 (electronic supplementary material, figure S2). Generally, while rates were faster when a younger age of crown-angiosperms was considered, different schemes resulted in contrasting rates for key branches. For example, the angiosperm stem lineage shows a faster rate of molecular evolution when a more ancient origin is inferred (SM4 and SM5), whereas when crown-angiosperms are estimated to be of Early Cretaceous or Late Jurassic age (SM1 and SM2), the molecular rate is slower (figure 3). Conversely, the branch leading to eudicots shows a slow rate under SM4 and SM5 but becomes 4–5 times faster under SM1 and SM2 (figure 3). Heterogeneity among branches in clock rate is accommodated in relaxed clock models by the 𝜎^2^ parameter, with larger values indicating greater variation among branches and thus violation of strict clock assumption. Across all partitions, we observed that younger estimates for the age of crown-angiosperms coincided with greater heterogeneity in clock rate among branches (figure 3).

## 4. Discussion

During all analyses across different calibration strategies, the effective prior exceeded the uniform calibration density (figure 2), compatible with the probability tail associated with each of the soft maximum calibration constraints. Furthermore, for all calibration strategies bar SM5 (*Cordaixylon iowensis*), the posterior age estimates exceeded both the uniform calibration density and the effective prior (figure 2). However, the extent to which the posterior exceeded the prior was not dependent on the age of the maximum, with the greatest exception associated with calibration strategy SM3 (*Phyllites* sp.).

The implementation of differing soft maximum age constraints had a large effect on the estimates of the angiosperm crown group age (figure 2). The younger maximum constraints produced younger estimates for the crown group, with the youngest (SM1, tricolpate pollen) estimating an origin for crown-angiosperms during the Early Cretaceous (129.4–130.5 Ma, Hauterivian). All other constraints produced estimates that exceeded the age of the earliest Cretaceous angiosperm pollen. A maximum age constraint derived from the Barremian (SM2, Monosulcate pollen) produced a Late Jurassic (145.0–155.3 Ma, Kimmeridgian–Tithonian) divergence time estimate for the angiosperm crown, while the maximum based on the Stonesfield Slate (SM3, *Phyllites* sp.) resulted in an Early–Middle Jurassic (180.2–164.8 Ma, Toarcian–Callovian) divergence date. The calibration expanded to include the Late Triassic pollen (SM4, *Retisulcites*) resulted in a Late Triassic–latest Permian estimate (250.6–201.9 Ma, Changhsingian–Norian), while the angiosperm total-group-based maximum age constraint (SM5; *Cordaixylon iowensis*) resulted in an Early Permian–Late Triassic (225.5–286.5 Ma, Artinskian–Norian) estimate for the age of crown-angiosperms. Thus, increasingly inclusive calibrations yielded estimates with less precision, reflected in broader highest posterior density intervals. The younger calibration densities resulted in estimates for the crown age constrained to just 0.6 (SM1) and 10.3 (SM2) Myr. More permissive calibrations also appear less informative, with the crown age estimated within 15.4 (SM3), 30.7 (SM4) and 61 (SM5) Myr.

### 4.1. Molecular clock methods can accommodate a Cretaceous angiosperm diversification

The results of our analyses demonstrate that contemporary relaxed molecular clock methods are compatible with interpretations of the fossil record [6,7,16,18]. If a crown-angiosperm affinity can be decisively rejected for all pre-Cretaceous plant remains, the soft maximum age constraint on the age of crown-angiosperms estimates either an Early Cretaceous or Late Jurassic age for the origin of crown-angiosperms (figure 2). However, accepting increasingly older equivocal records of crown-angiosperms in the formulation of the crown-angiosperm calibration results in concomitantly older estimates for the age of the angiosperm crown. The differences between the timescales arising from the different calibrations are substantial, far greater than the impact of different clock models, taxon sampling strategies or partitioning schemes [7,10,14]. In each case, the specified maximum age for crown-angiosperms affected the inferred rates of molecular evolution across the phylogeny and multiple genomes. This variation in rate was reflected in and accommodated by the clock parameter 𝜎^2^ that permits the relaxation of a strict clock. This shows that, with regard to the age of angiosperms, the most important aspect of a molecular clock analysis is the choice and justification of fossil calibrations. In almost all instances, the upper bound on both the effective prior and the posterior age estimate exceeds the oldest unequivocal angiosperm fossils. This does not imply conflict between molecular clock methods and the fossil record but, rather, reflects the expectation that crown-angiosperms diverged prior to the oldest known crown-angiosperm fossils.

### 4.2. Resolving the debate over the timing of angiosperm diversification

**Figure 4.**
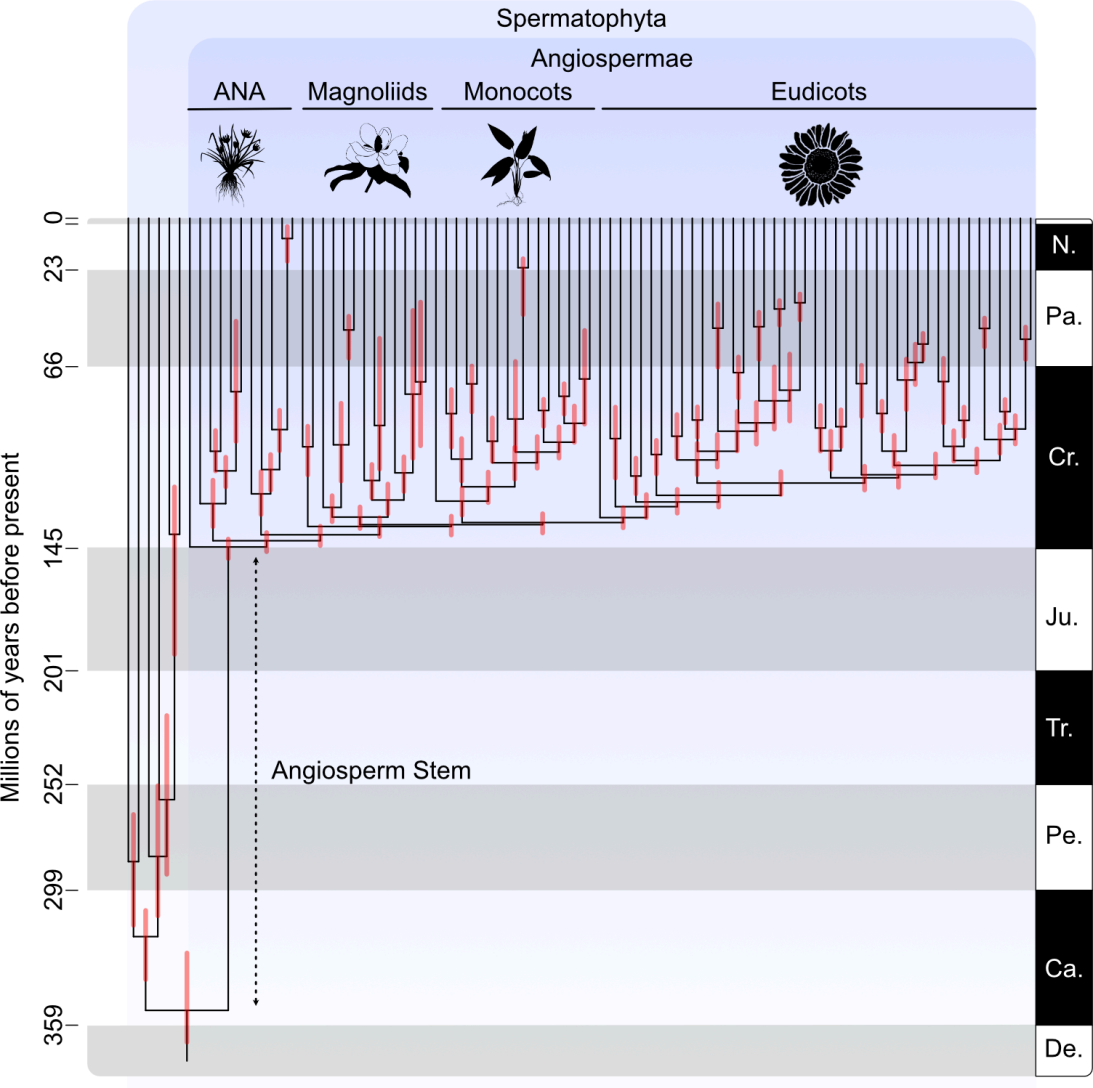
The timescale of angiosperm evolution estimated using the best-justified calibration scheme (SM2), where the maximum age of crown-angiosperms is constrained by the earliest appearances of monosulcate pollen in the Valanginian. A dotted line demonstrates the inferred length (and gap) of the angiosperm stem. Images were obtained from PhyloPic and are in the public domain.

The principal basis of uncertainty in the timing of the diversification of crown-angiosperms evidently lies in the interpretation of a small number of key fossils. As such, progress may be made through their reinterpretation. In particular, research into the wall ultrastructure of Anisian angiosperm-like pollen [25,44] would help to resolve its phylogenetic affinity. The age and provenance of *Phyllites* sp. from the Middle Jurassic Stonesfield Slate now seem to be unquestionable [24], but it is possible that further insights into its anatomy and, therefore, affinity may be leveraged through biogeochemical analysis, polarized light imaging [55] or elemental mapping [56]. However, the claims of crown-angiosperm affinity for these fossils are largely speculative, and so it is perhaps inappropriate that they are used to constrain molecular clock calibrations on the timing of divergence of crown-angiosperms; the low probability of their crown-angiosperm affinity is readily encompassed by the soft tail distribution on a maximum constraint defined on the earliest (Valanginian) occurrence of monosulcate pollen (calibration strategy SM2 above). It is our view that the timescale inferred with calibration strategy SM2, which infers a Kimmeridgian–Tithonian (145.0–155.3 Ma) divergence of crown-angiosperms, is the most appropriate conclusion based on the available data and methods (figure 4).

### 4.3. A Cretaceous origin of angiosperms exchanges one ‘Jurassic gap’ for another

The disparity between the fossil record of crown-angiosperms and molecular estimates for their time of origin was coined the ‘Jurassic gap’ [13], though some molecular estimates suggest that this gap could extend from the Permian through the Jurassic [57]. Our results show that the extent of this gap depends on the interpretation of the fossil record; it disappears altogether when calibrations are bounded by a decisive rejection of pre-Cretaceous crown-angiosperm records (figure 2). At face value, this may seem appealing, except that it serves only to create another ‘Jurassic gap’, this time of stem-angiosperms.

The oldest unequivocal fossil remains of acrogymnosperms are Late Carboniferous (312.38 Ma), and molecular estimates suggest a Late Devonian–Early Carboniferous (370–335 Ma; [27,58,59]) divergence from angiosperms. Thus, both fossil minima and molecular estimates indicate a 230–190 Myr interval between the origin of total-group and crown-angiosperms. Such gaps are also observed in the case of conifers and Gnetales within gymnosperms, though neither is as egregious as in angiosperms. Various fossil lineages have been resolved phylogenetically as stem-angiosperms, including glossopterids, Bennettitales, Petriellales, Caytoniales and Pentoxylales [60–64]. With the exception of Pentoxylales, the majority of these lineages originate during the Permian or Triassic, and the proposal that any might form the sister lineage to angiosperms remains controversial [65]. Thus, even if we were to resolve fully the angiosperm stem lineage—a currently optimistic prospect—a Jurassic gap in the evolutionary history of angiosperms will persist. This is significant because the stem-lineage is composed of fossil taxa that record the sequential assembly of crown-group characters. Angiosperms are marked by a suite of reproductive and vegetative innovations, including the closed carpel, endosperm and sieve elements, which are combined with characters found across other seed plants, such as vessels, reduced gametophytes and broad leaf architecture [66]. Resolving the stem lineage is then crucial to constraining hypotheses on the developmental evolution of the angiosperm body plan and the subsequent angiosperm terrestrial revolution [1]. Furthermore, the resolution of the angiosperm stem informs on the timing of the origin of crown-angiosperms by providing a biogeographic, ecological and taphonomic control on interpreting the absence of crown-angiosperm fossils in Early Cretaceous and pre-Cretaceous strata (cf. [12]). Thus, we argue that insights into the origin of crown-angiosperms are more likely to be found through the population and resolution of the angiosperm stem.

## Data Availability

Electronic supplementary material is available online [67]. All data and input files are available at [68].
